# Different phenotypes of microglia in animal models of Alzheimer disease

**DOI:** 10.1186/s12979-022-00300-0

**Published:** 2022-10-08

**Authors:** Yun Wei, Xianxiao Li

**Affiliations:** 1 Xiyuan Hospital of China Academy of Chinese Medical Sciences, 100091 Beijing, China; 2 Jingxi Cancer Hospital, 100161 Beijing, China

**Keywords:** Microglia phenotype, Microglia, Alzheimer disease, Neurodegeneration

## Abstract

Microglia are immune-competent cells that are critically involved in maintaining normal brain function. A prominent characteristic of Alzheimer disease (AD) is microglial proliferation and activation concentrated around amyloid plaques in the brain. Recent research has revealed numerous microglial phenotypes related to aging and AD, apart from the traditional M1 and M2 types. Redox signalling modulates the acquisition of the classical or alternative microglia activation phenotypes. The numerous microglial functions can be achieved through these multiple phenotypes, which are associated with distinct molecular signatures.

## Introduction

Microglia are the resident immune cells of the central nervous system (CNS) [1] and are crucially involved in maintaining brain homeostasis. Microglia are an extremely heterogeneous group of cells that interact with other CNS cells, including neurons, astrocytes, and oligodendrocytes [2]. Moreover, microglial morphology differs in the presence of neuronal cell bodies, dendrites, axons, myelinated axons, and blood vessels; a direct link between phenotypes and functions is not always straightforward. Microglial proliferation and activation concentrated around amyloid plaques is a prominent characteristic of Alzheimer disease (AD) [3]. Microglia regulate neuronal development and synaptic plasticity during learning and environmental adaptation, suggesting a crucial role in AD pathogenesis. Microglial activation in the CNS is heterogeneous and can be categorized as resting/surveilling, pro-inflammatory, and anti-inflammatory activation states, which depend on redox states. Recent single-cell RNA sequencing (RNA-seq) has revealed numerous aging- and AD-related microglial phenotypes, including “disease-associated microglia” (DAM), which is probably involved in protective phagocytosis, and “neurodegenerative microglia” (MGnD), which is a dysfunctional microglial phenotype. Furthermore, proliferative-region-associated microglia (PAM) arise during development and express genes enriched in DAM.

The microglial phenotype could be constantly shaped by the microenvironment in a time- and context-dependent manner. Herein, we discuss the relationship between distinct microglial phenotypes and AD.

## Microglia in the brain

Microglia are self-renewing and long-lived immune cells in the brain. Adult microglia are derived from unique yolk sac-primitive macrophages at embryonic days 8.5–9 in mice and migrate to the CNS during embryogenesis[4]. However, an alternative hypothesis suggests that microglial progenitors distinct from yolk sac-derived cells may exist and this hypothesis is receiving some support of many studies[5–7]. Microglia are mainly found in neurogenic niches; specifically, the subventricular and subgranular zone[8]. Under physiological conditions microglia self-renew over an organism’s entire lifespan. Microglia are relatively long-lived and rarely proliferate in the brain, except during certain CNS insults, including stress, lesions, infectious diseases, normal aging, a diet lack in phytochemicals[9], nutritional and hormonal disorders[10], which affects CNS homeostasis and microglial physiology. Microglia are not uniformly distributed throughout the CNS[11]; a large number of microglia are observed in the hippocampal dentate gyrus, substantia nigra, and parts of the basal ganglia; moreover, the olfactory telencephalon in mice has the largest microglial population. Additionally, there are different microglial sizes and ramification patterns within and between different histological layers of the cerebellar cortex. The microglial proportion ranges from 5% in the cortex and corpus callosum to 12% in the substantia nigra[12]. Regional heterogeneity of microglia could be attributed to the residential environment, especially interactions with neurons or neural progenitor cells; however, there could be intrinsic mechanisms contributing to the heterogeneity. After entering the brain, microglial progenitor cells quickly proliferate in situ during the first two postnatal weeks, with approximately 95% of microglia being generated[13]. The speed of microglial processes also is significantly slowed with age. Microglia may have an epithelioid, rod, amoeboid, multinucleated, or “dystrophic” morphological appearance. During early brain development, microglial cells present an amoeboid profile characterized by large and rounded cell bodies and short and thick branches. During the mature development stages, microglia present an amoeboid form characterized by fewer or shorter branched processes[14].

There has been active research on microglial function under pathological and physiological conditions. Microglia have a small cell body with highly branched processes; moreover, the microglial number and function are tightly controlled by the local microenvironment and interactions with surrounding cells under normal physiological conditions. Microglia enter the developing brain very early before neurogenesis, neuronal migration, myelination in various brain areas, as well as gliogenesis in the regional neuroepithelia[15]. Microglia were assumed to play a passive or supportive role in neurons; however, they are now known to be very motile within the CNS and to engage with the other brain’s intrinsic immune cells, neuronal circuit development, synaptic pruning, myelin turnover, neuronal excitability control, etc. Microglial-depleted mice show defective learning and memory formation abilities. However, the range of microglial functions remains unclear.

Although microglia are considered to be crucially involved in the pathogenesis and progression of numerous neurodegenerative disorders, their role remains unclear.

## Microglia in Alzheimer disease

AD is an age-related, progressive, and irreversible neurodegenerative disorder characterized by progressive loss of memory and cognitive abilities; moreover, it is the most common dementia form in the elderly [16]. AD is divided into familial (FAD) and sporadic forms. Neuroimmune inflammation and oxidative stress play an important role in the pathogenesis of AD. Microglia are major sources of free radicals such as superoxide nitric oxidein the brain, and play crucial roles in AD[17]. Numerous sporadic AD risk genes[18], including apolipoprotein E (ApoE), triggering receptor expressed on myeloid cells 2 (TREM2), Cluster differentiation(CD) 33, membrane-spanning 4-domains, subfamily A, member 6 A (MS4A6A), ATP-binding cassette, sub-family A, member 7 (ABCA7), and complement receptor 1 (CR1), are highly expressed in microglia and affect microglial phagocytosis of amyloid-beta peptides(Aβ). During the early and advanced AD stage, microglia protect against amyloid accumulation and promote neuropathology, respectively.

The role of microglia in neurodegenerative diseases has been intensively studied. Approximately two-thirds of novel AD-risk single nucleotide polymorphisms are exclusively[19] or dominantly expressed in microglia. Furthermore, AD involves increased microglial proliferation. In AD, microglia have a “double-edged sword” effect involving neurotoxic or neuroprotective functions based on contextual factors and disease stage[20]. During the early AD stage, microglia are involved in Aβ and tau clearance from the brain. Contrastingly, in the later AD stage, sustained microglial activation causes a chronic pro-inflammatory state, including increased production of pro-inflammatory cytokines, reactive oxygen species (ROS), and dysfunctional lysosomal deposits, which adversely affects neuronal survival and causes direct neuronal damage, concurrently promoting protein aggregation. This suggests that microglia are potential therapeutic targets for AD.

## Microglial phenotypes in AD

The restoration of redox balance may be determinant in driving microglia back to the resting state. Impairment of redox homeostasis possibly associated with microglia activation and then changes of microglial phenotypes. Historically, microglial phenotypes can be categorized according to the cell morphological features[12]. Microglial morphological changes are widely utilized to quantify microglial activation. Generally, highly ramified and amoeboid cells are designated as “resting” and “activated,” respectively, with a range of intermediate activation states (e.g., “intermediately activated,” “bipolar,” “rod-like,” “hypertrophied,” and “bushy”). Therefore, understanding the modulation of microglial phenotypic shifts and redox signaling is crucial for developing therapeutic strategies for AD.

## The classical microglia activation phenotypes

**Fig. 1:**
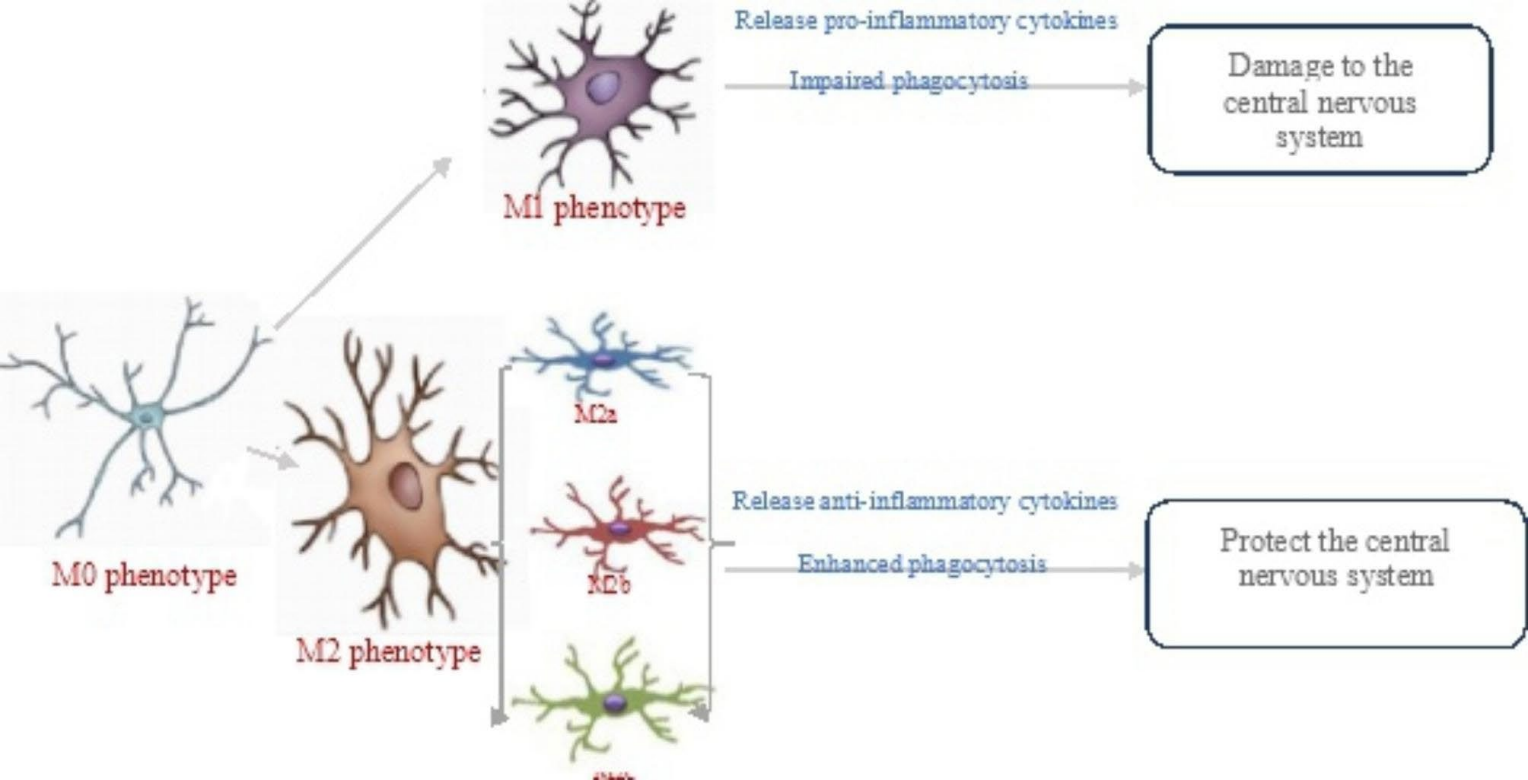
Functions of activated microglia in the M0/M1/M2 phenotype

Microglial activation has been considered as heterogeneous, with categorization into the M0, M1, and M2 phenotypes involved in surveillance, pro-inflammatory response, and anti-inflammatory response, respectively. M1 and M2 microglia are common in various neurodegenerative diseases, including AD, and could facilitate the conceptualization of microglia activities in vitro (see Table 1; Fig. 1).

### M0 phenotype

Microglial cells were considered to be in a “resting” state under healthy conditions[21], which is characterized by functional dormancy; expired, low, or absent expression associated with activation molecules, and an “immobile” branching morphology. In the healthy CNS, resting microglia present a low or no antigen-presenting cell phenotype. Resting microglia constantly extend and retract their thin ramified processes to monitor their surroundings for signs of infection or homeostasis-perturbing events; moreover, they remodel neural circuits by forming synaptic communications with adjacent neurons in healthy brains[22]. The microglial gene signature in mice revealed an association of loss of homeostatic microglial function with the degree of neuronal cell loss. The role of resting microglia remains unclear. However, this term is no longer unpopular since these “resting” cells are far from resting and exhibit restless and vigorous movement in their delicate ramified processes even in the normal CNS[23].

### M1 phenotype

M1 is a pro-inflammatory phenotype involved in neuroinflammatory processes[24]. M1-activated microglia can produce ROS due to reduce nicotinamide adenine dinucleotide phosphate oxidase activation (respiratory burst) and increase production of pro-inflammatory cytokines, including tumor necrosis factor alpha (TNF-α) and interleukin (IL)-1β, and then induce inflammation cascade [9]. Similar to macrophage activation, pro-inflammatory cytokine production is essential for microglial polarization toward the M1 phenotype. M1 microglia are considered as both beneficial and detrimental, depending on the temporal and regional characteristics of microglial activation.

### M2 phenotype

M2 polarization is also referred to as “alternative activation.” It is characterized by the release of anti-inflammatory cytokines and neurotrophins and is mainly involved in reparative and restorative processes while resolving acute inflammatory events. M2 polarization, which secretes anti-inflammatory cytokines and nutrient factors for promoting repair, regeneration, and restoring homeostasis, is widely considered as neuroprotective. Because microglia/macrophages respond to IL-4 or IL-13, M2 microglia show increased phagocytosis and produce growth factors, including insulin-like growth factor-1 (IGF-1), and anti-inflammatory cytokines, including IL-10. In M2 polarization, there are three described subpopulations because a single cohesive M2 phenotype cannot reflect the diversity of microglial populations[25]. M2a (alternative activation) is involved in repair and regeneration through the expression of anti-inflammatory and neurotrophic factors [26]; however, this phenotype is rare in AD brains [27]. The M2b transitional state (type II alternative activation) is involved in immune responses, with the expression of pro-inflammatory and anti-inflammatory mediators; contrastingly, the M2c state (acquired deactivation) mediates myelin debris clearance[28], neuroprotection, and release of anti-inflammatory cytokines[26].

### Intermediate polarization “M1½” state [29]

Several studies have suggested an intermediate state of polarization (“M1½”) exist in microglia and no clear lesion stage-dependent polarization patterns have been identified.

There is much overlap among M1½ and M1, M2; therefore, intermediate phenotype absent the unique markers and were observed in close proximity to blood vessels and tau pathology[30]. Normal resting microglia, reactive microglia, intermediate and bumpy forms and macrophage-like cells can be distinguished by Ionized calcium binding adaptor molecule 1 (IBA1), CD68, CD16 and CD163 and CD11b, CD45, c-musculoaponeurotic fibrosarcoma(c-MAF), and CD98 can be used further categorized(31).

**Table 1:** Differences of microglia in the M0/M1/M2 phenotype

	Possible markers		Morphological appearance	Activation pathway	Function	Secreted factors	Effect of fatty acids on microglia	Chemokines	Main energy source
M0 (Homeostatic microglia)	CD11b, P2ry12, Cx3cr1, Tmem119, Sall1, Tgfbr1, Fcrls, Hexb, Mertk, Gpr34, Olfml3, Siglech [32]		Branched, have small somata and multiple fine processes [33])	–	Have housekeeping functions and typically express genes involved in synaptic pruning, remodeling, and phagocytosis [34]	Soluble factors, including TNF-α and BDNF	–	CCL2	–
M1 (pro-inflammatory microglia)	CD86, CD16/32, MHC-11, CD40, IFN-γ CD11b, CCL2 iNOS, Cox-2, IL‐1β, IL‐6, TNF‐α [35, 36]		Larger somata and still branched compared with M0	LPS and IFN-γ [37]	Pro-inflammatory and pro-killing functions	TNF-α, IL-6, IL-12, IL-1β, IL-18, CXCL10, NO, ROS, iNOS, and proteolytic enzymes (MMP9, MMP3) [38]	saturated fatty acids increase the pro-inflammatory microglial phenotype	CCL2、CCL3、CCL4、CCL5、CXCL1、CXCL8、CXCL9、CXCL10	Preferentially use glycolysis [39]
M1½	co-expressing M1 makers and M2 markers [40]		Bipolar-shaped microglia [41]	--	improve cognition [42]	--	--	--	--
M2 (anti-inflammatory microglia) [43, 44]	M2a	CD206, SRs Arg1, Fizz-1 , YM1, CCL22	Amoeboid morphology [45]	IL-4 or IL-13 [46]	Involved in phagocytosis, tissue restoration, and tissue regeneration	IL-10, IGF-1, and trophic polyamines	Unsaturated fatty acids influence the anti-inflammatory microglial phenotype [46]	CCL22、CXCL8、CXCL12、CX3CL1	Oxidative phosphorylation and fatty acid oxidation [39]
	M2b	CD86, MHC II TNF-α, IL-6, IL-10, COX2		TLRs, FCγ and IL-1 receptors [47]	Involved in the recruitment of regulatory T cells	TNF-α, IL-1β, IL-10, and IL-6			
	M2c	SLAM, CD206 TGFβ, IL-10, CD163 [48]		IL-10, TGF-β, and glucocorticoid hormones, sphingosine kinase, and CD163. A membrane-bound receptor for haptoglobin/hemoglobin complexes [49]	Involved in anti-inflammatory and therapeutic functions	TGF-β, IL-10, sphingosine lipid kinase			

Regarding neurodegenerative diseases, transcriptome data have revealed that microglia express neurotoxic and neuroprotective factors, as well as genes involved in stress responses, misfolded proteins, and neuronal damage. The microglial M1/M2 balance is modulated by the redox status [50]. M1 and M2 categories can facilitate the illustration of the pathobiology of inflammatory and degenerative CNS disorders. Although functional classification of microglia as neurotoxic (M1) or neuroprotective (M2) has allowed conceptualization of microglial activities in vitro, further distinction provides a useful framework for exploring the diverse functions of the innate immune system in disease pathogenesis. Notably, M0-to-M1-to-M2 polarization involves changes in morphological appearance, quantity, and function. It remains unclear whether this represents a transition between different microglial types or a cycle of different states of the same microglial type. However, the M1/M2 distinction is still considered because it was developed through in vitro experiments in a simplified environment that cannot be replicated in vivo.

## The alternative microglia activation phenotypes

Microglial cells undergo morphological and functional changes in AD brains, which could extend beyond the M1/M2 dichotomy. Recent reports have indicated that during aging and age-related neurodegenerative diseases, microglial phenotypes are transient and demonstrate temporal and spatial evolution. Microglia can change from homeostatic to other reactive states, including activated response microglia (ARM), DAM, MGnD, lipid droplet-accumulating microglia (LDAM), and dark microglia(DM).

### Lipid droplet-accumulating microglia (LDAM) [51]

With aging, microglia accumulate lipid droplets. Lipid droplets can form due to various environmental and cellular conditions, including increased levels of extracellular lipids, inflammatory events, increased ROS levels, and intracellular metabolic changes. According to lipidomics analysis, lipid droplets mainly contain glycerolipids, with low cholesteryl ester levels. LDAM, which recedes amyloid plaque formation [52] and accounts for > 50% of all microglia in the aged hippocampus [53]. However, microglia without lipid droplets lack typical age-related functional impairments and a primed phenotype, suggesting that LDAM may be the primary detrimental microglial in the aging brain.

LDAM, which differ from DAM and MGnD with respect to the transcriptional profile, have defective phagocytosis, produce high ROS levels, and secrete pro-inflammatory cytokines. Several genes regulate the LDAM phenotype, including progranulin gene (GRN), solute carrier family 33 member 1(SLC33A1), sorting nexin 17(SNX17), vacuolar protein sorting retromer complex component, Niemann-Pick disease type C 2(NPC2), and neuronal ceroid lipofuscinosis 3(NCL3), with some genes overlapping between DAM and MGnD [54]. LDAM produce high levels of ROS and reactive nitrogen species, release higher levels of cytokines (e.g., IL-6, CCL3, and CXCL10) under control conditions, and show defective phagocytosis. Typical aging genes, including AXL, C-type lectin domain family 7 member A (CLEC7A), and CYBB, are regulated in a reciprocal direction in LDAM. Furthermore, TREM2 and APOE, which are involved in neurodegeneration, are upregulated in DAM and MGnD but are not regulated in LDAM. In the aging brain, LDAM showed severe phagocytosis deficits compared with microglia without lipid droplets. Therefore, this phenotype can be considered a dysfunctional microglia state.

### Disease-associated microglia (DAM)

Upregulated genes in DAM, also known as Aβ-plaque-associated microglia, include Axl, Apoe, Clec7a, Itgax, Galectin 3 (LGALS3), and cystatin F (CST7), which are considered to be involved in neurodegenerative diseases. DAM are Trem2-dependent microglia observed with aging, AD, frontotemporal dementia, and amyotrophic lateral sclerosis. Most DAM genes do not directly accelerate neurodegeneration; however, a few, including APOE, may be involved in deteriorating neurodegeneration [55].

Single-cell RNA-seq identified three microglial subpopulations in AD: homeostatic, intermediate, and DAM. DAM comprise two clusters, namely, DAM1 and DAM2, which have distinct patterns of core genes. DAM1 core genes include β2-microglobulin (B2M), Apoe, and Dap12/Tyrobp; contrastingly, DAM2 is characterized by increased lipoprotein lipase (LPL), Cst7, Trem2, Clec7a, and Axl expression [56]. The transition from the normal state to DAM1 is TREM2-independent while shifting from DAM1 to DAM2 requires TREM2 signals. Although DAM microglia are known to be associated with Aβ plaques, it remains unclear whether they have a protective or disease-inducing function. Srinivasan et al. compared the transcriptional profile of human AD microglia (HAM) and DAM in the mouse 5xFAD model, the results suggest that there is little resemblance between the DAM profile and HAM profile [57].

### Neurodegenerative microglia (MGnD)

The MGnD phenotype, which is very similar to DAM, has been observed in several neurodegenerative models and is induced by phagocytosis of apoptotic neurons, which is activated by Aβ plaques and AAV-P301L-tau injection. M0-to-MGnD polarization is associated with enhanced phagocytosis of plaques, apoptotic neurons, and neuritic dystrophy in APP-PS1 mice [58] and human AD [59]). MGnD microglia strongly expressed an extracellular vesicle (EV) marker and interferon-related genes, which indicated increased synthesis of EVs. EV release in MGnD microglia is over thrice that in homeostatic microglia [58]. This MGnD phenotype switch is triggered by TREM2, which activates inflammatory genes (including Axl, Itgax, Clec7a, and Apoe) and subsequently suppresses the homeostatic microglial phenotype (including Tgfb(r), Hexb, P2ry12, and Cx3cr1) and TFs (including Mef2a, Mafb, and Sall1). There is a partial overlap between the M1 and MGnD microglial phenotypes. Furthermore, it remains unclear whether MGnD microglia are ultimately beneficial or harmful in AD.

### Dark microglia (DM)

DM are increased in non-homeostatic conditions (e.g., AD pathology, aging, fractalkine signaling deficiency, and chronic stress) [60] and during normal brain development; however, they are rare in healthy young adult mice. DM is morphologically characterized by disrupted mitochondria, dilation of the Golgi apparatus and endoplasmic reticulum, and cell shrinkage [61]. The electron density of DM could be attributed to the particular nature and distribution of their cytoplasmic and nucleoplasmic proteins and lipids, especially those with high affinity for osmium tetroxide [62]. Alternatively, DM may be newly formed microglial cells yet to spread to their designated areas [63]. DM exhibit a highly activated phenotype with strong CD11b and TREM2 expression, as well as extensive encircling of synaptic clefts in the presence of amyloid deposits [64]. However, DM does not express CD11c. DM is involved in the pathological remodeling of neuronal circuits.

### Proliferative-region‐associated microglia (PAM)

PAM [65] mainly occur in the developing corpus callosum and cerebellar white matter during a transient period in the first postnatal week in aging and AD models [66]. PAM have amoeboid morphology, are metabolically active, and phagocytose newly formed oligodendrocytes. They share a characteristic gene signature with DAM [67]. However, unlike DAM, PAM appearance is independent of the TREM2-APOE axis [67]. PAM express numerous trophic factors, including Igf1, secreted phosphoprotein 1(Spp1), Lgals1, and Lgals3, as well as distinct cytokine/chemokine secretion profiles, including Ccl3 and Ccl4. Accordingly, PAM may be predominant in areas showing myelination during development or in dysmyelinating disorders.

### White matter–associated microglia (WAMs) [68]

White matter (WM) primarily comprises myelinated axons connecting neurons in various brain regions. WM damage or abnormalities can occur in various neuronal diseases, including AD. After WM damage, microglia are activated to multiple forms depending on several conditions, including their proximity to the lesion, location within the brain, and disease type. WM is rich in lipids and devoid of neuronal ligands that control microglial activity, including CX3CL1, CD47, and CD20040. WAMs partly share the DAM gene signature (Apoe, B2m, Lyz2, and Clec7a) and are characterized by the activation of genes implicated in phagocytic activity and lipid metabolism. WAMs are dependent on TREM2 signaling and aging. In aging, microglia activation appears to be shifted toward the WAM state, whereas in AD, the shift is toward DAMs. These findings suggest that WAM are precursors to DAM [69].

### Activated response microglia (ARM)

Reactive microgliosis with neuroinflammation is a neuropathological hallmark of the AD brain [70]. ARM comprise specialized subgroups that overexpress MHC type II and putative tissue repair genes (Dkk2, Gpnmb, and Spp1) and are strongly enriched with AD risk genes. There is a partial overlap between ARM and DAM; however, ARM are detected in the brains of young wild-type mice. Therefore, ARM could be found in the normal aging brain.

### Interferon-response microglia (IRM)

The interferon (IFN) pathway is exclusively activated in Clec7a + microglia concomitantly with the MGnD program. Clec7a + microglia show increased expression of MHC I antigen (H2-D1), H2-K1, Tap1, Tap2, Tapbp, and B2m. Moreover, Clec7a + microglia upregulate IFN-stimulated genes that function as costimulatory molecules, including Cd72, Cd180, and Cd274 (i.e., Pd-l1). IFN can directly activate microglia and induce a pro-inflammatory response, which partially overlaps with, but is distinct from, the DAM/MGnD program. IRM express an IFN-related gene module and are increased in brains with β-amyloid pathology distinct from that of DAM cells [71].

### Necroptotic microglia (NM)

NM are highly pro-inflammatory and immunogenic in AD. Necroptosis, which is a novel cell death pathway, was originally considered as dependent on the kinase RIPK1; however, it is now known to be dependent on RIPK3 and the mixed lineage kinase domain-like protein (MLKL) [72]. NM is among the key players in neuroinflammation and neurodegeneration [73]. However, it has been recently demonstrated that [74] necrosome formation may trigger a robust, cell death-independent, pro-inflammatory response. Therefore, the role of NM in AD remains unclear.

### Late-response microglia (LRM)

Hansruedi et al. reported that LRM are exclusively observed in the later AD stages in a mouse model (CK-p25) [75]. There is a substantial similarity between the expression profiles of LRM and DAM. LRM show increased expression of numerous genes upregulated in DAM (e.g., Cd9, Itgax, Clec7a, Cd63, Spp1, Fth1, Axl, Lpl, Cst7, Ctsb, and Apoe). However, these two microglial states may be different because numerous antiviral and IFN response genes, including antiviral and interferon response genes, as well as MHC class II components [75], are significantly upregulated in LRM but not in DAM.

### Dystrophic microglia

The term dystrophic is derived from morphological changes demonstrated by studies on brain sections. Microglial dystrophy appears to precede neurofibrillary degeneration. Although dystrophic microglia are frequently identified in neurodegenerative diseases, including AD, it remains unclear whether it has a causal role. Dystrophic microglia could induce impairment of neuronal activity in both aging and diseased states probably because of store iron through elevated levels of ferritin [76]. Experts have found microglial dystrophy are the characterize neurodegenerative changes in AD, but not microglial activation [77].

**Table 2:** Features of Newly-described microglial phenotypes

	Distinctive Features	unique phenotypic properties	phenotypic markers	Function	Incentives	Formation	Content	Distribution	Immunoreactivity	Animal models
Typical microglia	The resident non-activated microglia present ramified morphology with long extension; however, upon activation, they retract the extensions and become amoeboid [18]	--	TMEM119, CD11B, and P2RY12/P2RY13 expression, with low CD45 expression [78]	Regulate synaptic plasticity, learning, and memory mechanisms [79]	No mention	Erythromyeloid progenitor cells in the embryonic yolk sac [80], with the same origin as that of peripheral macrophages (myeloid progenitors)	Comprise 5–10% of all CNS cells [81]	in all brain regions, mainly in the gray matter [82]	High immunoreactivity for homeostatic markers (GFP in CX3CR1-GFP mice, P2RY12, IBA1, etc.)	---
LDAM	Containing large inclusions, i.e., lipofuscin granules [83]	Exhibit a unique transcriptional signature, show phagocytosis deficits, and produce increased levels of ROS and pro-inflammatory cytokines [51]	GRN, solute carrier family 33 Member 1 (SLC33A), SNX17, vacuolar protein sorting retromer complex component (VPS35), NPC2, and CLN3 [54]	Represent a dysfunctional and pro-inflammatory microglia state in the aging brain [51]	Increased extracellular lipid levels, inflammatory events, increased ROS levels, and intracellular metabolic changes [84]	Age-related neuroinflammation [51]	Approximately 50% of microglia in the aging brain accumulate lipid droplets [51]	Frequently found in the hippocampus and thalamus [51]	Immunoreactive for Iba1, Plin2, Plin3	Five human familial AD mutations (5XFAD) [85]
DAM	Rounded enlarged bodies, with 45% of DAMs being dystrophic [86]	upregulated expression of genes related to lipid metabolism, phagocytosis, and AD pathology, downregulated expression of homeostatic markers, without changes in expression of inflammatory cytokines [67]	anti-inflammatory DAM genes (e.g., Kcnj2, Nceh1, Timp2, CXCR4), pro-inflammatory DAM-specific gene (e.g.: Ptgs2/Cox2 or Tlr2, CD14, CD44)	Compared with normal microglia, DAMs show progressively increased lipid metabolism and expression of phagocytic genes, and therefore protect against AD and clear Aβ [67]	Accumulation of danger molecules present on apoptotic bodies of dying neural cells, lipid degradation products, and myelin debris [87]	Dependent on TREM2 signaling [67], also partially by APOE	A proportion of DAM increases with aging, accounting for 3% of all microglial cells in 20-month-old mice [67].	Within the cortex, but not the cerebellum, of AD mice [40]	Immunoreactive for IBA1 and HLA-DR	PS19 tau transgenic, SOD1-G93A transgenic, and aged mice; five human familial AD mutations (5XFAD) and CK-p25; PS2APP and APP/PS1 [67, 88]
MGnD	A phagocytic microglia phenotype with reduced ramifications and cell volume [89]	The signature genes associated with MGnD regulate lipid metabolism and phagocytosis [54]	Strong downregulated expression of homeostatic genes, with upregulated expression of selective genes including Spp1, Itgax, Axl, Clec7a, Lgals3, Apoe, and Grn [59]	Protective and represent an initial response to neuronal injury	Induced by neuronal apoptosis or Aβ plaques [59]	The switch from homeostatic microglia to MGnD is regulated by the TREM2-APOE pathway [59]	No mention	In APP-PS1 mice, MGnD primarily encircled amyloid plaques and dystrophic neurites, which are blanketed by homeostatic microglia in the periphery.	Low or no immunoreactivity for P2RY12	APP-PS1 mice, APP-PS1 *Trem2*−/− mice [59, 67]
Dark microglia	Electron microscopy reveals condensed cytoplasm and nucleoplasm, increased projections to synapses, and increased encircling of axon terminals and dendritic spines [90]	a downregulated expression of homeostatic markers, CX3CR1, IBA1 and P2RY12, but strongly expressed the microglia-specific 4D4 in their processes [62]	Strongly express CD11b, Trem246, and 4D4, with downregulated expression of IBA1, CX3CR1, and P2RY12 [64]	Extensively engulfing dendritic spines, axon terminals, and entire synapses	Chronic stress, aging, fractalkine signaling deficiency (CX3 CR1 knockout mice), and Alzheimer’s disease pathology (APP-PS1 mice) [64]	Derived from yolk sac, brain progenitors, or bone marrow-derived cells recruited to the brain in a CCR2‐independent manner [91]	In age-matched APP/PS1 littermates, the number of dark microglia corresponded to almost two‐thirds of the typical microglial population [64]	The hippocampal CA1 region (strata lacunosum-molecular and radiatum), subgranular layers of the cerebral cortex, basolateral nucleus of the amygdala, and hypothalamic median eminence [64]	Low immunoreactivity for homeostatic markers (GFP in CX3CR1-GFP mice, IBA1, CD11b, 4D4, TREM2); no ALDH1L1, OLIG2, P2RY12, 4C12, MHCII, CD206, CD11c expression [64]	APPSwe-PS1ΔE9、CX3CR1 knockout mice [64, 92]
PAM	Compared with typical microglia, PAM are amoeboid with thicker primary branches and larger cell bodies [93]	enrich many metabolic genes including almost the entire molecular machineries for oxidative phosphorylation, glycolysis and beta oxidation	Characterized by expression of numerous trophic factors including Igf1, Spp1, Lgals1, and Lgals3 [63]; contrastingly, many genes (Apoe, Igf1, Lilrb4, Lyz2, Colec12, Msr1, Map1lc3b) have upregulated expression [94]	Phagocytose newly formed oligodendrocytes and possibly new-born astrocytes during development	No mention	Independent of the TREM2-APOE axis [65]	Comprise one-third of the normal microglial population in APP-PS1 mice [64]	Developing cerebellar white matter and corpus callosum	Immunoreactive for CLEC7A	*Trem2*−/− or *Apoe*−/− [65]
WAM	Cluster in nodules	upregulated genes linked to atherosclerosis, cytokine signaling, and apoptosis [68], downregulation of genes expressed in homeostatic microglia, such as checkpoint genes	Increased expression of the NF-κB pathway and adhesion family GPCR GPR56 (ADGRG1) [95]	May represent a potentially protective response [68]	Aging and cerebral hypoperfusion	Dependent on age and TREM2 but not APOE signaling [68]; APOE dependent [96]	~ 20% in 21-month-old AD model mice	White matter tracts from the corpus callosum [68]	Immunoreactive for CD68 [96]	MTX rats (MTX through intraperitoneal injection every week at a dose of 200 mg/kg/week for a total of 4 weeks, and a final dose of 800 mg/kg) (97); Apoe−/− mice; APP/PS1 mouse model [68, 98]
ARM [70, 97]	No mention	MHC-II presentation (Cd74, Ctsb, Cstd), inflammatory processes (Cst7, Clec7a, Itgax) putative tissue repair genes (Dkk 1, Spp1, Gpnmb) and AD risk genes like APOE	Increased expression of histocompatibility complex class II genes (Cd74, H2-Ab1, and H2-Aa) and pro-inflammatory genes Cst7, Clec7a, and Itgax (encoding CD11c)	No mention	No mention	No mention	Increased from approximately ~ 3% in 3-month-old mice to ~ 12% of the total microglia in 21-month-old mice	No mention	No mention	
IRM	Adopts a reactive morphology after rIFN-β exposure, with reduced dendrite length, branch, and terminal points [99]	the expression of thousands of interferon-stimulated genes [100]	Increased expression of Ifit3, Ifitm3, Irf7, and Oasl2 [100]	Contributing to the inflammatory tone of the aged and AD brain [71]	Aging, viral infection, cuprizone-induced demyelination	can be triggered by nucleic acid (NA)-containing plaques	15% of all APP/PS1 microglia [97]	No mention	Immunoreactive for IBA1	5XFAD mice [96], CK-p25 mice [75], APP-PS1, tauopathy (P301S和P301L)
NM	Chromosome fragmentation, membrane blebbing, and cell volume shrinkage	Release various pro-inflammatory cytokines and chemokines [101], including tumor necrosis factor-α and chemokine (C-C motif) ligand 2	--	Highly pro-inflammatory and immunogenic [72]	Different cellular stimuli, including TNF-α, FAS ligand, TRAIL, IFNγ, ischemia-reperfusion injury, and double-stranded RNA (dsRNA)	Through TLR4 activation	No mention	No mention	Immunoreactive for IBA1	
Dystrophic microglia	Characterized by a dystrophic morphology, including process deramification, shortening, gnarling and beading, spheroid formation, and cytoplasmic fragmentation [77], 102]	Significant changes in genes controlling inflammation, including the NF-κB signaling pathway, and upregulation of complement genes C3 and complement factor B	--	Impaired neuronal activity, iron storage, reduced phagocytosis, and increased ROS production	Aging, iron-fed [76]	Through iron accumulation	There was an increase in the proportion of dystrophic microglia with age. In the case of neurodegenerative pathology, approximately 45% of the microglia were found to be dystrophic.	Near sites of tau pathology and amyloid plaques, in the neocortical gray matter of layers II–III in aged chimpanzees [30, 103]	Immunoreactive for IBA1（104）	aged *Tupaia belangeri* (mean age 7.5 years), Binge Alcohol Model [104],aged marmosets [105], LPS (0.33 mg/kg) administration to adult rats [106]

In summary, redox imbalance in microglia plays a critical role in AD, and then microglia express different phenotypes. According to research findings [107], NADPH oxidase (NOX2) -mediated redox signalling modulates the acquisition of the classical or alternative microglia activation phenotypes by regulating major transcriptional programs mediated through nuclear factor-κB(NF-κB) and NF- E2 p45-related factor 2 (Nrf2). However, the activation patterns of pro-inflammatory “M1” or immunoregulatory “M2” phenotypes overlap. In the newly described microglial phenotypes, the LDAM transcriptional signature showed almost no overlap with DAM, MGnD, and DM. Notably, typical aging genes, including AXL, CLEC7A, and CYBB, are regulated in a reciprocal direction in LDAM. Furthermore, TREM2 and APOE, which are crucially involved in the progression of neurodegeneration, were upregulated in DAM and MGnD but not in LDAM and PAM. PAM mirror the gene signature and function of DAM and MGnD subsets in disease conditions; specifically, they are associated with the phagocytosis of newly formed and dying oligodendrocytes during normal post-natal development, as well as defective phagocytosis, high ROS levels, and secretion of pro-inflammatory cytokines.

Additionally, DAM, ARMs, and dystrophic microglia can be discovered during the normal brain aging process. LDAM may be the primary detrimental microglia state in the aging brain. while DAM are actively phagocytic populations, LDAM are severely impaired in this function. Further, the microglial phenotype is strongly associated with the disease course. Gene changes in microglia in the late AD stage are similar to those in DAM, IRM, LRM, while PAM mainly in the early AD stages. The microglial phenotypes may drastically change throughout AD progression or may be driven by diverse stimuli (see Table 2).

Microglia phenotypes are characterized by both molecular characteristics of microglia and increased or decreased expression of genes. These genetic changes promote the microglia phenotype transformation. These different phenotypes are likely to be continuous cell identities that can blend with each other. This indicates the need for tracking with fine temporal resolution to capture the full spectrum of microglia cell states.

## Conclusion

Microglia are critically involved in maintaining normal brain function. But it remains unclear whether microglia are beneficial, detrimental, or both in AD progression. Microglia are considered to have various reaction states, with a tremendous shift from the previously used M1/M2 classification. Although the M1 and M2 categories can allow conceptualization of microglia activities in vitro, microglia rarely display a significant bias toward either the M1 or M2 phenotype. During aging and in AD, microglia acquire different phenotypes, which dependent on redox status. Restoration of redox balance may be determinant in driving microglia back to the resting state [108]. Accordingly, mastering the stage-specific switching of microglial phenotypes within appropriate time windows may inform improved therapeutic strategies. However, it remains unclear how the transcriptional programs in microglia cells change over time as they transition from their initial homeostatic state in the healthy brain to the reactive phenotypes seen in neurodegeneration.

## Data Availability

All articles referenced in this review are available.

## Abbreviations

AD: Alzheimer’s disease.
Aβ: amyloid-beta peptides.
ApoE: apolipoprotein E.
ARM: activated response microglia.
CD: Cluster differentiation.
CLEC7A: C-type lectin domain family 7 member A.
CNS: central nervous system.
DAM: disease-associated microglia.
EV: extracellular vesicle.
HAM: human AD microglia.
IFN: interferon.
IL: Interleukin.
LDAM: Lipid droplet-accumulating microglia.
LRM: Late-response microglia.
MGnD: neurodegenerative microglia.
NM: Necroptotic microglia.
PAM: proliferative-region-associated microglia.
ROS: reactive oxygen species.
TNF-α: tumor necrosis factor alpha.
TREM2: triggering receptor expressed on myeloid cells 2.
WM: White matter.

## References

[1] Ana Badimon HJ, Strasburger P, Ayata X, Chen,Aditya Nair, A, Ikegami P, Hwang AT, Chan SM, Graves, Joseph O, Uweru C, Ledderose MG, Kutlu MA, Wheeler A, Kahan M, Ishikawa Y-C, Wang, Yong-Hwee E, Loh JX, Jiang D, James Surmeier SC, Robson WG, Junger R, Sebra ES, Calipari PJ, Kenny UB Eyo, Marco Colonna, Francisco J. Quintana, Hiroaki Wake, Viviana Gradinaru, and Anne Schaefer. Negative feedback control of neuronal activity by microglia. Nature. 2020; 586(7829): 417–423. doi: 10.1038/s41586-020-2777-8. 10.1038/s41586-020-2777-8 PMC7577179 32999463

[2] Kettenmann H, Kirchhoff F, Verkhratsky A (2013). Microglia: new roles for the synaptic stripper. Neuron.

[3] David V, Hansen JE, Hanson M, Sheng (2018). Microglia in Alzheimer’s disease. J Cell Biol.

[4] Florent Ginhoux M, Greter M, Leboeuf S, Nandi P, See S, Gokhan MF, Mehler SJ, Conway L, Ng G, Stanley ER, Igor M, Samokhvalov (2010). Miriam Merad. Fate mapping analysis reveals that adult microglia derive from primitive macrophages. Science.

[5] Xu J, Zhu L, He S, Wu Y, Jin W, Yu T, Qu JY, Wen Z (2015). Temporal-Spatial Resolution Fate Mapping Reveals Distinct Origins for Embryonic and Adult Microglia in Zebrafish. Dev Cell.

[6] Askew K, Li K, Olmos-Alonso A, Garcia-Moreno F, Liang Y, Richardson P, Tipton T, Chapman MA, Riecken K, Beccari S, Sierra A, Molnár Z, Cragg MS, Garaschuk O, Perry VH, Gomez-Nicola D (2017). Coupled Proliferation and Apoptosis Maintain the Rapid Turnover of Microglia in the Adult Brain. Cell Rep.

[7] Shrutokirti De D, Van Deren E, Peden M, Hockin A, Boulet S, Titen (2018). Mario R Capecchi. Two distinct ontogenies confer heterogeneity to mouse brain microglia. Development.

[8] Nora Hagemeyer K-M, Hanft M-A, Akriditou N, Unger, Eun S, Park ER, Stanley O, Staszewski L, Dimou (2017). Marco Prinz. Microglia contribute to normal myelinogenesis and to oligodendrocyte progenitor maintenance during adulthood. Acta Neuropathol.

[9] Giovanni Brunetti GD, Rosa M, Scuto M, Leri M, Stefani C, Schmitz-Linneweber (2020). Vittorio Calabrese, and Nadine Saul4. Healthspan Maintenance and Prevention of Parkinson’s-like Phenotypes with Hydroxytyrosol and Oleuropein Aglycone in C. elegans. Int J Mol Sci.

[10] Bobbi Fleiss J, Van Steenwinckel C, Bokobza IK, Shearer (2021). Emily Ross-Munro, Pierre Gressens. Microglia-Mediated Neurodegeneration in Perinatal Brain Injuries. Biomolecules.

[11] Sophie Miquel C, Champ J, Day E, Aarts BA, Bahr M, Bakker D, Bánáti V, Calabrese T, Cederholm J, Cryan L, Dye JA, Farrimond A, Korosi S, Layé S, Maudsley D, Milenkovic MH, Mohajeri J, Sijben A, Solomon, Jeremy PE, Spencer (2018). Sandrine Thuret, Wim Vanden Berghe, David Vauzour, Bruno Vellas, Keith Wesnes, Peter Willatts, Raphael Wittenberg, Lucie Geurts. Poor cognitive ageing: Vulnerabilities, mechanisms and the impact of nutritional interventions. Ageing Res Rev.

[12] Ochocka N, Kaminska B (2021). Microglia Diversity in Healthy and Diseased Brain: Insights from Single-Cell Omics. Int J Mol Sci.

[13] Alliot F, Godin I, Pessac B (1999). Microglia derive from progenitors, originating from the yolk sac, and which proliferate in the brain. Brain Res Dev Brain Res.

[14] Yun-Long Tan Y, Yuan L (2020). Microglial regional heterogeneity and its role in the brain. Mol Psychiatry.

[15] David A, Menassa (2018). Diego Gomez-Nicola. Microglial Dynamics During Human Brain Development. Front Immunol.

[16] Juan Ramón, Perea (2020). Marta Bolós, and Jesús Avila. Microglia in Alzheimer’s Disease in the Context of Tau Pathology. Biomolecules.

[17] Takahiro A, Kato F, Hyodo M, Yamato H, Utsumi S, Kanba (2015). Redox and microglia in the pathophysiology of schizophrenia. Yakugaku Zasshi.

[18] Al-Khalifah MAl-Onaizi,A, Qasem D, Ayman, ElAli (2020). Role of Microglia in Modulating Adult Neurogenesis in Health and Neurodegeneration. Int J Mol Sci.

[19] Amanda, McQuade (2019). Mathew Blurton-Jones. Microglia in Alzheimer’s Disease: Exploring How Genetics and Phenotype Influence Risk. J Mol Biol.

[20] Suzanne Hickman S, Izzy P, Sen L, Morsett (2018). Joseph El Khoury. Microglia in neurodegeneration. Nat Neurosci.

[21] Naoki Abe T, Nishihara T, Yorozuya, Tanaka J (2020). Microglia and Macrophages in the Pathological Central and Peripheral Nervous Systems. Cells.

[22] Dorothy P, Schafer EK, Lehrman AG, Kautzman R, Koyama AR, Mardinly R, Yamasaki RM, Ransohoff ME, Greenberg BA, Barres, Beth Stevens (2012). Microglia sculpt postnatal neural circuits in an activity and complement-dependent manner. Neuron.

[23] Nimmerjahn A, Kirchhoff F, Helmchen F (2005). Resting microglial cells are highly dynamic surveillants of brain parenchyma in vivo. Science.

[24] Ma Y, Wang J, Wang Y, Yang GY (2017). The biphasic function of microglia in ischemic stroke. Prog Neurobiol.

[25] Franco R, Fernández-Suárez D (2015). Alternatively activated microglia and macrophages in the central nervous system. Prog Neurobiol.

[26] Zhang L, Zhang J, You Z (2018). Switching of the Microglial Activation Phenotype Is a Possible Treatment for Depression Disorder. Front Cell Neurosci.

[27] Douglas G (2015). Walker and Lih-Fen Lue. Immune phenotypes of microglia in human neurodegenerative disease: challenges to detecting microglial polarization in human brains. Alzheimers Res Ther.

[28] Mecha M, Yanguas-Casás N, Feliú A, Mestre L, Carrillo-Salinas F, Azcoitia I, Yong VW, Guaza C (2019). The endocannabinoid 2-AG enhances spontaneous remyelination by targeting microglia. Brain Behav Immun.

[29] Nelson H, Knudsen C-H, Lee. Identity Crisis: CD301b(+) Mononuclear Phagocytes Blur the M1-M2 Macrophage Line. Immunity. 2016;45(3):461–463. doi: 10.1016/j.immuni.2016.09.004. 10.1016/j.immuni.2016.09.004 27653596

[30] Melissa K, Edler CC, Sherwood RS, Meindl E, Munger WD, Hopkins JJ, Ely JM, Erwin Daniel P, Perl EJ, Mufson PR (2018). Hof, and Mary Ann Raghanti. Microglia changes associated to Alzheimer’s disease pathology in aged chimpanzees. J Comp Neurol.

[31] Annovazzi L, Mellai M, Bovio E, Mazzetti S, Pollo B, Davide, Schiffer (2018). Microglia immunophenotyping in gliomas. Oncol Lett.

[32] Susan Brandenburg A, Blank AD, Bungert, Vajkoczy P (2021). Distinction of Microglia and Macrophages in Glioblastoma: Close Relatives, Different Tasks?. Int J Mol Sci.

[33] Rodríguez JJ, Witton J, Olabarria M, Noristani HN, Verkhratsky A (2010). Increase in the density of resting microglia precedes neuritic plaque formation and microglial activation in a transgenic model of Alzheimer’s disease. Cell Death Dis.

[34] Oluwaseun Fatoba T, Itokazu T, Yamashita (2020). Microglia as therapeutic target in central nervous system disorders. J Pharmacol Sci.

[35] Shin J-H, Hwang YS, Jung B-K, Seo S-H, Ham D-W (2021). Eun-Hee Shin. Reduction of Amyloid Burden by Proliferated Homeostatic Microglia in Toxoplasma gondii-Infected Alzheimer’s Disease Model Mice. Int J Mol Sci.

[36] Liu Li-QiangLXiang-Rong, Zhao J-Y, Yan F, Wang R-L, Wen S-H, Wang L, Luo Y-M (2018). Xun-Ming Ji. Brain-selective mild hypothermia promotes long-term white matter integrity after ischemic stroke in mice. CNS Neurosci Ther.

[37] Yosuke Kumamoto, Joao Paulo G, Camporez MJ, Jurczak M, Shanabrough T, Horvath GI, Shulman A, Iwasaki (2016). CD301b(+) Mononuclear Phagocytes Maintain Positive Energy Balance through Secretion of Resistin-like Molecule Alpha. Immunity.

[38] Sophie C, Gray KJ, Kinghorn, Nathaniel S, Woodling (2020). Shifting equilibriums in Alzheimer’s disease: the complex roles of microglia in neuroinflammation, neuronal survival and neurogenesis. Neural Regen Res.

[39] Sheng Yang C, Qin Z-W, Hu L-Q, Zhou H-H, Chen YM, Bosco DB, Wang W, Wu L-J (2021). Dai-Shi Tian. Microglia reprogram metabolic profiles for phenotype and function changes in central nervous system. Neurobiol Dis.

[40] Kumar A, Alvarez-Croda DM, Stoica BA, Faden AI, Loane DJ (2016). Microglial/Macrophage Polarization Dynamics following Traumatic Brain Injury. J Neurotrauma.

[41] Zheng ZV, Lyu H, Lam SYuE, Lam PK, Poon WS, George Kwok Chu Wong (2020). The Dynamics of Microglial Polarization Reveal the Resident Neuroinflammatory Responses After Subarachnoid Hemorrhage. Transl Stroke Res.

[42] Sam Famenini EA, Rigali HM, Olivera-Perez J, Dang MT, Chang R, Halder RV, Rao M, Pellegrini (2017). Verna Porter, Dale Bredesen, Milan Fiala. Increased intermediate M1-M2 macrophage polarization and improved cognition in mild cognitive impairment patients on ω-3 supplementation. FASEB J.

[43] Chhor V, Le Charpentier T, Lebon S, Oré MV, Celador IL, Josserand J, Degos V, Jacotot E, Hagberg H, Sävman K, Mallard C, Gressens P, Fleiss B (2013). Characterization of phenotype markers and neuronotoxic potential of polarised primary microglia in vitro. Brain Behav Immun.

[44] Houan Tu H, Chu S, Guan F, Hao N, Xu Z, Zhao Y, Liang. The role of the M1/M2 microglia in the process from cancer pain to morphine tolerance.Tissue Cell. 2021; 68:101438. doi: 10.1016/j.tice.2020.101438. 10.1016/j.tice.2020.101438 33220596

[45] Rock RB, Gekker G, Hu S, Sheng WS, Cheeran M, Lokensgard JR (2004). Peterson P. K. Role of microglia in central nervous system infections. Clin Microbiol.

[46] Wynn TA, Chawla A, Pollard JW (2013). Macrophage biology in development, homeostasis and disease. Nature.

[47] Martinez FO, Gordon S. The M1 and M2 paradigm of macrophage activation: time for reassessment. F1000Prime Rep, 2014, 6: 13. doi: 10.12703/P6-13. 10.12703/P6-13 PMC3944738 24669294

[48] Song GJ, Suk K (2017). Pharmacological Modulation of Functional Phenotypes of Microglia in Neurodegenerative Diseases. Front Aging Neurosci.

[49] Wilcock DM (2014). Neuroinflammatory phenotypes and their roles in Alzheimer’s disease. Neurodegener Dis.

[50] Peter J, Murray JE, Allen SK, Biswas EA, Fisher DW, Gilroy S, Goerdt S, Gordon JA, Hamilton, Lionel B, Ivashkiv T, Lawrence M, Locati A, Mantovani FO, Martinez J-L, Mege, David M, Mosser G, Natoli, Jeroen P, Saeij, Joachim L, Schultze (2014). Kari Ann Shirey, Antonio Sica, Jill Suttles, Irina Udalova, Jo A van Ginderachter, Stefanie N Vogel, Thomas A Wynn. Macrophage activation and polarization: nomenclature and experimental guidelines. Immunity.

[51] Julia Marschallinger,Tal, Iram M, Zardeneta SE, Lee B, Lehallier MS, Haney JV, Pluvinage V, Mathur O, Hahn DW, Morgens J, Kim J, Tevini TK, Felder H, Wolinski CR, Bertozzi MC, Bassik (2020). Ludwig Aigner, and Tony Wyss-Coray. Lipid droplet accumulating microglia represent a dysfunctional and pro-inflammatory state in the aging brain. Nat Neurosci Nat Neurosci.

[52] Praticò D, Uryu K, Leight S, Trojanoswki JQ, Lee VM (2001). Increased lipid peroxidation precedes amyloid plaque formation in an animal model of Alzheimer amyloidosis. J Neurosci.

[53] Jung ES, Mook-Jung I (2020). New Microglia on the Block. Cell Metab.

[54] Afridi R, Lee W-H, Suk K (2020). Microglia Gone Awry: Linking Immunometabolism to Neurodegeneration. Front Cell Neurosci.

[55] Akira Sobue O, Komine Y, Hara F, Endo H, Mizoguchi S, Watanabe S, Murayama T, Saito TC (2021). Saido,Naruhiko Sahara, Makoto Higuchi, Tomoo Ogi, and Koji Yamanaka. Microglial gene signature reveals loss of homeostatic microglia associated with neurodegeneration of Alzheimer’s disease. Acta Neuropathol Commun.

[56] Tina Schwabe K, Srinivasan H (2020). Shifting paradigms: The central role of microglia in Alzheimer’s disease. Neurobiol Dis.

[57] Karpagam Srinivasan BA, Friedman A, Etxeberria MA, Huntley, Marcel P, van der Brug O, Foreman JS, Paw Z, Modrusan, Thomas G, Beach, Geidy E, Serrano (2020). David V Hansen. Alzheimer’s Patient Microglia Exhibit Enhanced Aging and Unique Transcriptional Activation. Cell Rep.

[58] Kevin Clayton JC, Delpech S, Herron N, Iwahara M, Ericsson T, Saito TC, Saido S, Ikezu, Tsuneya, Ikezu (2021). Plaque associated microglia hyper-secrete extracellular vesicles and accelerate tau propagation in a humanized APP mouse model. Mol Neurodegener.

[59] Krasemann S, Madore C, Cialic R, Baufeld C, Calcagno N, Fatimy RE, Beckers L, Xu EO’Loughlin,Y, Fanek Z, Greco DJ, Scott T, Smith, George Tweet, Zachary Humulock, Tobias Zrzavy, Patricia Conde-Sanroman, Mar Gacias, Zhiping Weng, Hao Chen, Emily Tjon, Fargol Mazaheri, Kristin Hartmann, Asaf Madi, Jason Ulrich, Markus Glatzel, Anna Worthmann, J, Heeren B, Budnik C, Lemere T, Ikezu FL, Heppner, Vladimir Litvak, DM, Holtzman H, Lassmann HL Weiner, Jordi Ochando, Christian Haass, and Oleg Butovsky. The TREM2-APOE pathway drives the transcriptional phenotype of dysfunctional microglia in neurodegenerative diseases. Immunity. 2017; 47(3): 566–581.e9. doi: 10.1016/j.immuni.2017.08.008. 10.1016/j.immuni.2017.08.008 PMC5719893 28930663

[60] St-Pierre MK, Šimončičová E, Bögi E, Tremblay M (2020). Shedding Light on the Dark Side of the Microglia. ASN Neuro.

[61] Vassilis, Stratoulias. Jose Luis Venero, Marie-Ève Tremblay, and Bertrand Joseph. Microglial subtypes: diversity within the microglial community. EMBO J. 2019 Sep 2; 38(17): e101997. doi: 10.15252/embj.2019101997. 10.15252/embj.2019101997 PMC6717890 31373067

[62] Kanchan Bisht K, Sharma B, Lacoste, Marie-Ève T (2016). Dark microglia: Why are they dark?. Commun Integr Biol.

[63] Nichols MR, Wendeln Marie-KimSt-Pierre,Ann-Christin, Nyasha J, Makoni LK, Gouwens EC, Garrad M, Sohrabi, Jonas J, Neher (2019). Marie-Eve Tremblay, Colin K Combs. Inflammatory mechanisms in neurodegeneration. J Neurochem.

[64] Bisht K, Sharma KP, Lecours C, Sánchez MG, El Hajj H, Milior G, Olmos-Alonso A, Gómez-Nicola D, Luheshi G, Vallières L, Branchi I, Maggi L, Limatola C, Butovsky O, Tremblay M (2016). Dark microglia: A new phenotype predominantly associated with pathological states. Glia.

[65] Li Q, Cheng Z, Zhou Lu, Darmanis S, Neff NF, Okamoto J, Gulati G, Bennett ML, Lu O, Sun LE, Clarke J, Marschallinger G, Yu SR, Quake (2019). Tony Wyss-Coray, Ben A Barres. Developmental Heterogeneity of Microglia and Brain Myeloid Cells Revealed by Deep Single-Cell RNA Sequencing. Neuron.

[66] Kang SS, Ebbert MTW, Baker KE, Cook C, Wang X, Sens JP, Kocher JP, Petrucelli L, Fryer JD (2018). Microglial translational profiling reveals a convergent APOE pathway from aging, amyloid, and tau. J Exp Med.

[67] Keren-Shaul H, Spinrad A, Weiner A, Matcovitch-Natan O, Dvir-Szternfeld R, Ulland TK, David E, Baruch K, Lara-Astaiso D, Toth B, Itzkovitz S, Colonna M, Schwartz M, Amit I (2017). A Unique Microglia Type Associated with Restricting Development of Alzheimer’s Disease. Cell.

[68] Shima Safaiyan S, Besson-Girard T, Kaya L, Cantuti-Castelvetri Lu, Liu H, Ji M, Schifferer G, Gouna F, Usifo N, Kannaiyan D, Fitzner X, Xiang, Moritz J, Rossner (2021). Matthias Brendel, Ozgun Gokce, Mikael Simons. White matter aging drives microglial diversity Neuron.

[69] Roseborough AD, Jaremek VM, Whitehead SN (2022). Editorial Focus: White matter-associated microglia (WAMs) represent an important link between aging, white matter disease and microglial activity. Geroscience.

[70] De Strooper B, Karran E (2016). The Cellular Phase of Alzheimer’s Disease. Cell.

[71] Friedman BA, Srinivasan K, Ayalon G, Meilandt WJ, Lin H, Huntley MA, Cao Y, Lee SH, Haddick PCG, Ngu H, Modrusan Z, Larson JL, Kaminker JS, van der Brug MP, Hansen DV (2018). Diverse Brain Myeloid Expression Profiles Reveal Distinct Microglial Activation States and Aspects of Alzheimer’s Disease Not Evident in Mouse Models. Cell Rep.

[72] Silke J, Rickard JA, Gerlic M (2015). The diverse role of RIP kinases in necroptosis and inflammation. Nat Immunol.

[73] Yang S-H, Lee DK, Shin J, Lee S, Baek S, Kim J, Jung H, Hah J-M (2017). YoungSoo Kim. Nec-1 alleviates cognitive impairment with reduction of Aβ and tau abnormalities in APP/PS1 mice. EMBO Mol Med.

[74] Weinlich R, Oberst A, Beere HM, Green DR (2017). Necroptosis in development, inflammation and disease. Nat Rev Mol Cell Biol.

[75] Hansruedi Mathys C, Adaikkan F, Gao, Jennie Z, Young E, Manet M, Hemberg PL, De Jager RM, Ransohoff A, Regev (2017). Li-Huei Tsai. Temporal Tracking of Microglia Activation in Neurodegeneration at Single-Cell Resolution. Cell Rep.

[76] Boyd Kenkhuis A, Somarakis L, de Haan O, Dzyubachyk ME, IJsselsteijn, Noel FCC, de Miranda, Boudewijn PF, Lelieveldt J, Dijkstra, Willeke MC, van Roon-Mom (2021). Thomas Höllt, Louise van der Weerd. Iron loading is a prominent feature of activated microglia in Alzheimer’s disease patients. Acta Neuropathol Commun.

[77] Wolfgang J, Streit H, Braak Q-S, Xue (2009). Ingo Bechmann. Dystrophic (senescent) rather than activated microglial cells are associated with tau pathology and likely precede neurodegeneration in Alzheimer’s disease. Acta Neuropathol.

[78] Elizabeth C, Wright-Jin DH, Gutmann (2019). Microglia as Dynamic Cellular Mediators of Brain Function. Trends Mol Med.

[79] Augusto-Oliveira M, Arrifano GP, Lopes-Araújo A, Santos-Sacramento L, Takeda PY, Anthony DC, Malva JO, Crespo-Lopez ME (2019). What Do Microglia Really Do in Healthy Adult Brain? Cells.

[80] Ginhoux F, Lim S, Hoeffel G, Low D, Huber T (2013). Origin and differentiation of microglia. Front Cell Neurosci.

[81] Hannah Y, Collins CJB. Isolation and Culture of Rodent Microglia to Promote a Dynamic Ramified Morphology in Serum-free Medium. J Vis Exp. 2018; (133):57122. doi:10.3791/57122. 10.3791/57122 PMC5931678 29578519

[82] Ana I, Rojo G, McBean M, Cindric J, Egea MG, López P, Rada (2014). Neven Zarkovic, and Antonio Cuadrado. Redox Control of Microglial Function: Molecular Mechanisms and Functional Significance. Antioxid Redox Signal.

[83] Brawek B, Skok M, Garaschuk O (2021). Changing Functional Signatures of Microglia along the Axis of Brain Aging. Int J Mol Sci.

[84] Rambold AS, Cohen S, Lippincott-Schwartz J (2015). Fatty acid trafficking in starved cells: regulation by lipid droplet lipolysis, autophagy, and mitochondrial fusion dynamics. Dev Cell.

[85] Johanna M, Zirknitzer J, Unger MS, Poupardin R, Rieß T, Paiement N, Zerbe H (2021). Birgit Hutter-Paier, Herbert Reitsamer, Ludwig Aigner. The Leukotriene Receptor Antagonist Montelukast Attenuates Neuroinflammation and Affects Cognition in Transgenic 5xFAD Mice. Int J Mol Sci.

[86] Ryan K, Shahidehpour RE, Higdon NG, Crawford, Janna H, Neltner, Eseosa T, Ighodaro E, Patel D, Price, Peter T, Nelson, Adam D, Bachstetter (2021). Dystrophic microglia are associated with neurodegenerative disease and not healthy aging in the human brain. Neurobiol Aging.

[87] Aleksandra Deczkowska H, Keren-Shaul A, Weiner M, Colonna M, Schwartz I, Amit (2018). Disease-Associated Microglia: A Universal Immune Sensor of Neurodegeneration. Cell.

[88] Srikant Rangaraju EB, Dammer SA, Raza P, Rathakrishnan H, Xiao T, Gao DM, Duong MW, Pennington JJ, Lah, Nicholas T, Seyfried. Allan I Levey. Identification and therapeutic modulation of a pro-inflammatory subset of disease-associated-microglia in Alzheimer’s disease. 2018;13(1):24. doi: 10.1186/s13024-018-0254-8. 10.1186/s13024-018-0254-8 PMC5963076 29784049

[89] Hana, Yeh, and, Tsuneya, Ikezu (2019). Transcriptional and epigenetic regulation of microglia in health and disease. Trends Mol Med.

[90] Julie C, Savage (2018). Katherine Picard, Fernando González-Ibáñez, and Marie-Ève Tremblay. A Brief History of Microglial Ultrastructure: Distinctive Features, Phenotypes, and Functions Discovered Over the Past 60 Years by Electron Microscopy. Front Immunol.

[91] Bruttger J, Karram K, Wörtge S, Regen T, Marini F, Hoppmann N, Klein M, Blank T, Yona S, Wolf Y, Mack M, Pinteaux E, Müller W, Zipp F, Binder H, Bopp T, Prinz M, Jung S, Waisman A (2015). Genetic cell ablation reveals clusters of local self-renewing microglia in the mammalian central nervous system. Immunity.

[92] Hajj HE, Savage JC, Bisht K, Parent M, Vallières L (2019). Serge Rivest, Marie-Ève Tremblay. Ultrastructural evidence of microglial heterogeneity in Alzheimer’s disease amyloid pathology. J Neuroinflammation.

[93] Staszewski O, Hagemeyer N (2019). Unique microglia expression profile in developing white matter. BMC Res Notes.

[94] Ayata P, Badimon A, Strasburger HJ, Duff MK, Montgomery SE, Loh YE, Ebert A, Pimenova AA, Ramirez BR, Chan AT, Sullivan JM, Purushothaman I, Scarpa JR, Goate AM, Busslinger M, Shen L, Losic B, Schaefer A (2018). Epigenetic regulation of brain region-specific microglia clearance activity. Nat Neurosci.

[95] van der MarlijnPoel T, Ulas MR, Mizee C-C, Hsiao, Suzanne SM, Miedema A, Schuurman KG, Helder B, Sander W, Tas JL, Schultze (2019). Jörg Hamann, Inge Huitinga. Transcriptional profiling of human microglia reveals grey-white matter heterogeneity and multiple sclerosis-associated changes. Nat Commun.

[96] Roseborough AD, Jaremek VM, Whitehead SN (2022). Editorial Focus: White matter-associated microglia (WAMs) represent an important link between aging, white matter disease and microglial activity. GeroScience.

[97] Berlin C, Lange K, Lekaye HC, Hopland K, Phillips S, Piao J, Tabar V. Long-term clinically relevant rodent model of methotrexate-induced cognitive impairment. Neuro Oncol. 2020 Aug 17;22(8):1126–1137. doi: 10.1093/neuonc/noaa086. 10.1093/neuonc/noaa086 PMC7594568 32242229

[98] Roseborough AD, Jaremek VM, Whitehead SN (2021). Editorial Focus: White matter-associated microglia (WAMs) represent an important link between aging, white matter disease and microglial activity. Geroscience.

[99] Ethan R, Roy B, Wang Y-W, Wan G, Chiu A, Cole Z, Yin NE, Propson Y, Xu JL, Jankowsky Z, Liu, Virginia M-Y, Lee JQ, Trojanowski, Stephen D, Ginsberg (2020). Oleg Butovsky, Hui Zheng, Wei Cao. Type I interferon response drives neuroinflammation and synapse loss in Alzheimer disease. J Clin Invest.

[100] Timothy R, Hammond C, Dufort L, Dissing-Olesen S, Giera A, Young A, Wysoker AJ, Walker F, Gergits M, Segel J, Nemesh SE, Marsh, Arpiar Saunders, Evan Macosko, Florent Ginhoux, J, Chen RJM, Franklin X, Piao SA McCarroll, and Beth Stevens. Single cell RNA sequencing of microglia throughout the mouse lifespan and in the injured brain reveals complex cell-state changes. Immunity. 2019; 50(1): 253–271.e6. doi: 10.1016/j.immuni.2018.11.004. 10.1016/j.immuni.2018.11.004 PMC6655561 30471926

[101] Zhang Mengying Xu, Yiyue P, Tong Hu, Yue Y, Limpanont H, Ping K, Okanurak Wu, Yanqi P, Dekumyoy Z, Hongli D, Watthanakulpanich Wu, Zhongdao W, Zhi, Lv, Zhiyue (2017). Apoptosis and necroptosis of mouse hippocampal and parenchymal astrocytes, microglia and neurons caused by Angiostrongylus cantonensis infection. Parasit Vectors.

[102] Wolfgang J, Streit NW, Sammons, Amanda J, Kuhns D, Larry, Sparks (2004). Dystrophic microglia in the aging human brain. Glia.

[103] Dafina M, Angelova,David R (2019). Brown Microglia and the aging brain: are senescent microglia the key to neurodegeneration?. J Neurochem.

[104] Marshall SA, Justin A, McClain, Jessica I, Wooden KN. Microglia Dystrophy Following Binge-Like Alcohol Exposure in Adolescent and Adult Male Rats. Front Neuroanat. 2020;14:52. doi: 10.3389/fnana.2020.00052. eCollection 2020. 10.3389/fnana.2020.00052 PMC7439004 32903737

[105] Juan D, Rodriguez-Callejas E, Fuchs (2016). Claudia Perez-Cruz. Evidence of Tau Hyperphosphorylation and Dystrophic Microglia in the Common Marmoset. Front Aging Neurosci.

[106] Joana Costa d’Avila. Siqueira LD, Aurélien Mazeraud EP, Azevedo, Debora Foguel, Hugo Caire Castro-Faria-Neto, Tarek Sharshar, Fabrice Chrétien, Fernando Augusto Bozza. Age-related cognitive impairment is associated with long-term neuroinflammation and oxidative stress in a mouse model of episodic systemic inflammation. J Neuroinflammation. 2018;15(1):28. doi: 10.1186/s12974-018-1059-y. 10.1186/s12974-018-1059-y PMC5791311 29382344

[107] Vilhardt F, Haslund-Vinding J, Jaquet V, McBean G (2017). Microglia antioxidant systems and redox signalling. Br J Pharmacol.

[108] Nadia G, Innamorato I, Lastres-Becker A, Cuadrado (2009). Role of microglial redox balance in modulation of neuroinflammation. Curr Opin Neurol.

